# Crystal structure of 5-(furan-2-yl)-*N*-phenyl-1,3,4-oxa­diazol-2-amine

**DOI:** 10.1107/S2056989015019453

**Published:** 2015-10-24

**Authors:** Santosh Paswan, Manoj K. Bharty, Sanyucta Kumari, Sushil K. Gupta, Nand K. Singh

**Affiliations:** a Department of Chemistry, Banaras Hindu University, Varanasi 221 005, India; b School of Studies in Chemistry, Jiwaji University, Gwalior 474 011, India

**Keywords:** crystal structure, cyclized oxa­diazole derivative, hydrogen bonding, C—H⋯π inter­actions, π–π inter­actions

## Abstract

The title compound, C_12_H_9_N_3_O_2_, was obtained as a cyclized oxa­diazole derivative from substituted thio­semicarbazide in the presence of manganese(II) acetate. The furan ring is disordered over two orientations, with occupancies of 0.76 (2) and 0.24 (2). The dihedral angles between the central oxa­diazole ring and the pendant phenyl ring and furan ring (major disorder component) are 3.34 (18) and 5.7 (6)°, respectively. A short intra­molecular C—H⋯O contact generates an *S*(6) ring. In the crystal, inversion dimers linked by pairs of N—H⋯N hydrogen bonds generate *R*_2_^2^[8] loops. The dimers are linked by C—H⋯π and π–π inter­actions [range of centroid–centroid distances = 3.291 (2)–3.460 (8) Å], generating a three-dimensional network.

## Related literature   

For heterocyclic ligands that form metal complexes, see: Tarafder *et al.* (2001 ▸); Ali & Ali (2007 ▸); Singh *et al.* (2007 ▸); Zhao *et al.* (2007 ▸); Zhang *et al.* (2007 ▸); Amin *et al.* (2004 ▸). For applications in medicine and agriculture, see: Pachhamia & Parikh (1988 ▸); Xu *et al.* (2002 ▸). For related structures, see: Foks *et al.* (2002 ▸); Dani *et al.* (2013 ▸).
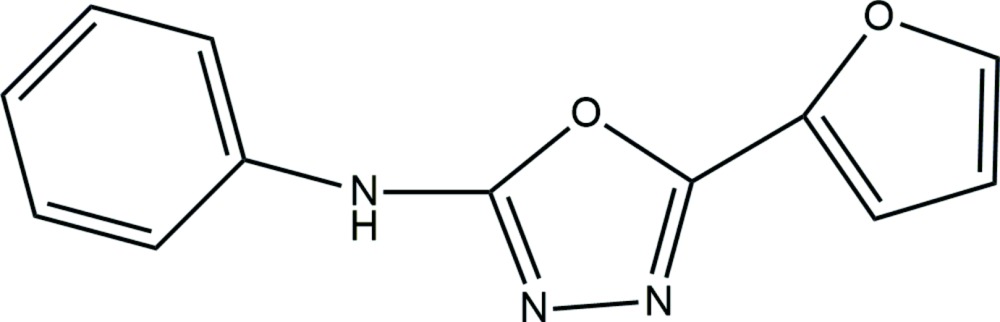


## Experimental   

### Crystal data  

C_12_H_9_N_3_O_2_
*M* *_r_* = 227.22
Monoclinic, 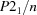

*a* = 13.195 (3) Å
*b* = 5.6162 (8) Å
*c* = 14.958 (3) Å
β = 107.00 (2)°
*V* = 1060.0 (3) Å^3^
*Z* = 4
Mo *K*α radiation
μ = 0.10 mm^−1^
*T* = 293 K
0.4 × 0.3 × 0.15 mm

### Data collection  

Agilent Xcalibur Eos diffractometer
Absorption correction: multi-scan (*CrysAlis PRO*; Agilent, 2011 ▸) *T*_min_ = 0.941, *T*_max_ = 1.000
4305 measured reflections
2405 independent reflections
1057 reflections with *I* > 2σ(*I*)
*R*_int_ = 0.039

### Refinement  

*R*[*F* ^2^ > 2σ(*F* ^2^)] = 0.064
*wR*(*F* ^2^) = 0.142
*S* = 1.01
2405 reflections
174 parameters
40 restraints
H atoms treated by a mixture of independent and constrained refinement
Δρ_max_ = 0.19 e Å^−3^
Δρ_min_ = −0.21 e Å^−3^

### 

Data collection: *CrysAlis PRO* (Agilent, 2011 ▸); cell refinement: *CrysAlis PRO*; data reduction: *CrysAlis PRO*; program(s) used to solve structure: *SHELXS2013* (Sheldrick, 2008 ▸); program(s) used to refine structure: *SHELXL2014* (Sheldrick, 2015 ▸); molecular graphics: *SHELXTL* (Sheldrick, 2008 ▸); software used to prepare material for publication: *SHELXTL*.

## Supplementary Material

Crystal structure: contains datablock(s) I. DOI: 10.1107/S2056989015019453/hb7522sup1.cif

Structure factors: contains datablock(s) I. DOI: 10.1107/S2056989015019453/hb7522Isup2.hkl

Supporting information file. DOI: 10.1107/S2056989015019453/hb7522Isup3.cml

**Fig. 1.**
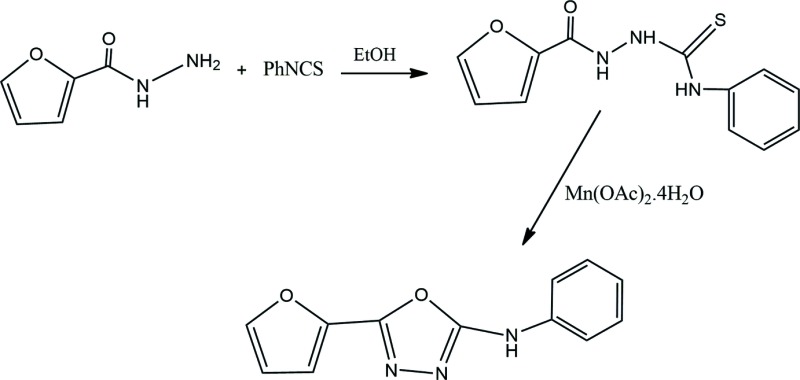
Scheme showing the synthesis of the title compound.

. DOI: 10.1107/S2056989015019453/hb7522fig1.tif
Scheme showing the synthesis of the title compound.

**Fig. 2.**
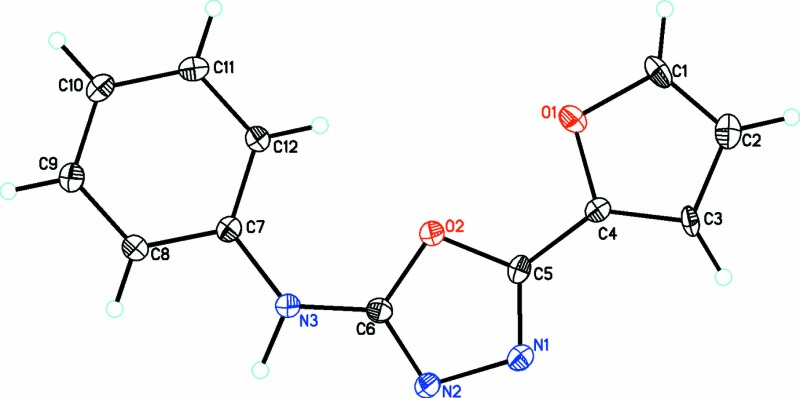
The molecular structure of (I) showing 50% probability displacement ellipsoids.

. DOI: 10.1107/S2056989015019453/hb7522fig2.tif
The mol­ecular structure of (I) showing 50% probability displacement ellipsoids.

**Fig. 3.**
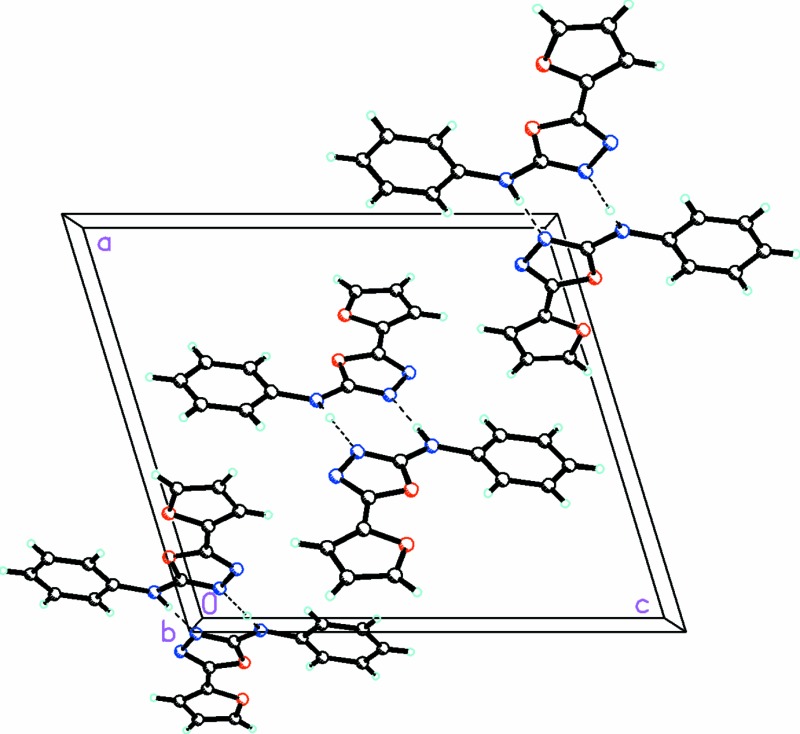
The molecular packing of the title compound, viewed along the b-axis. Dashed lines indicate weak N—H···N intermolecular hydrogen bonding between the oxadiazole ring and the amine group, forming dimers with an R22[8] ring motif.

Click here for additional data file.b . DOI: 10.1107/S2056989015019453/hb7522fig3.tif
The mol­ecular packing of the title compound, viewed along the *b*-axis. Dashed lines indicate weak N—H⋯N inter­molecular hydrogen bonding between the oxa­diazole ring and the amine group, forming dimers with an 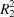
[8] ring motif.

CCDC reference: 1431289

Additional supporting information:  crystallographic information; 3D view; checkCIF report

## Figures and Tables

**Table 1:** Hydrogen-bond geometry (, ) *Cg*1 and *Cg*4 are the centroids of the O1*A*/C1/C2/C3/C4 and C7C12 five- and six-membered rings, respectively.

*D*H*A*	*D*H	H*A*	*D* *A*	*D*H*A*
N3H3*N*3N2^i^	1.02(3)	1.87(3)	2.892(3)	178(2)
C12H12*A*O2	0.93	2.27	2.892(4)	123
C9H9*A* *Cg*1^ii^	0.93	3.00	3.653(4)	129
C2H2*A* *Cg*4^iii^	0.93	2.93	3.664(5)	137

Symmetry codes: (i) 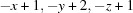; (ii) 
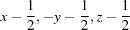
; (iii) 
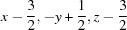
.

## supplementary crystallographic information

### S1. Comment

1,3,4-Oxa­diazole-2-thio­nes, an important class of heterocyclic ligands in the field of coordination chemistry (Tarafder *et al.*, 2001; Ali & Ali, 2007; Singh *et al.*, 2007; Zhao *et al.*, 2007; Zhang *et al.*, 2007; Amin *et al.*, 2004) have wide applications both in medicine and agriculture (Pachhamia & Parikh, 1988; Xu *et al.*, 2002). The cyclization of 3-acyl­dithio­carbazate esters, N-aroyldi­thio­carbaza­tes and their salts to the corresponding 1,3,4-oxa­diazole in the presence of base is reported in the literature (Foks *et al.*, 2002). Further, 5-benzyl-N- phenyl-1,3,4-thia­diazole-2-amine and 2-(5-phenyl-1,3,4-thia­diazol-2-yl)pyridine have also been reported in the presence of managnese(II) nitrate *via* loss of H_2_O (Dani *et al.*, 2013). It is known that in the presence of strong acid, N-acyl­hydrazine carbodi­thio­ate is converted into thia­diazole but in presence of weak acid or base they are cyclized into oxa­diazole. In the present case, managanese(II) acetate behaves like a weak acid and thus converts thio­semicarbazide into oxa­diazole *via* loss of H_2_S.

In the title compound (Fig. 2), the mean plane of the central oxa­diazole ring (O2/C5/N1/N2/C6) forms dihedral angles of 5.65 and 3.34° with the furan (O1/C1–C4) and phenyl rings (C7–C12), respectively. Both the furan and phenyl rings are twisted by an angle of 7.51°. The C–N bond lengths, N1–C5 1.290 (4) and N2–C6 1.302 (4) Å, are similar to standard C ═N 1.28 Å. The distances found within the oxa­diazole ring are inter­mediate between single and double bond, suggesting considerable delocalization in the ring. In the crystal, pairs of inter­molecular N—H···N hydrogen bonds between the oxa­diazole ring and the amine group forming dimers with an R^2^_2_[8] ring motif (Fig. 3, Table 1) and an intra­molecular C—H···O inter­action is also found. Molecules are further linked by weak C—H···N and two C—H···π inter­actions, involving (C7–C12) and (O1/C1–C4) rings. In addition, weak π···π inter­molecular stacking inter­actions [Cg1···Cg2 (x, 1+y, z) = 3.460 (8)Å; Cg2···Cg2 (1–x, 1–y, 1–z) = 3.291 (2)Å; Cg2···Cg3 (x, –1+y, z) = 3.431 (4)Å; Cg1: O1/C1–C4; Cg2: O2/C5/N1/N2/C6; Cg3: O1A/C1A–C4A] are present and influences the crystal packing. The furan ring is disordered over two positions, with occupancies of 0.76 (2) and 0.24 (2).

### S2. Experimental

Referring to Fig. 1, a mixture of furan-2-carb­oxy­lic acid hydrazide (1.260 g, 10 mmol) and phenyl iso­thio­cyanate (1.2 ml, 10 mmol) in absolute ethanol (20 mL) was refluxed for 4 h. The solid *N*-(furan-2-carbonyl)-4-phenyl­thio­semicarbazide obtained upon cooling was filtered off and washed with water and ether (50:50 v/v). A mixture of methano­lic solution of *N*-(furan-2-carbonyl)-4-phenyl­thio­semicarbazide (0.261 g, 1.00 mmol) and Mn(OAc)_2_.4H_2_O (0.246 g, 1 mmol) was stirred for 4 h. A clear yellow solution obtained was filtered off and kept for crystallization. Colourless needles were obtained after 10 days. Yield: 60%; m.p.: 476–478 K. Anal. Calc. for C_12_H_9_N_3_O_2_(%):C 63.43, H 3.99, N 18.49. Found: C 63.68, H 4.05, N 18.61. ^1^H NMR (DMSO-d_6_): δ [ppm] = 11.60 (s, 1H, NH), 8.80–7.31 (m, 3H, furan), 7.80–6.76 (m, 5H, Phenyl). ^13^C NMR (DMSO-d_6_): δ [ppm] = 161.3 (C6), 162.0 (C5), 153.7 (C4), 146.9 (C1), 144.8 (C3), 140.7 (C2) (furan C); 120.6–145.2 (phenyl C) (Fig. 2). IR (selected, KBr): 3210 [ν(N– H)], 1605 [ν(C═N)], 1076 [ν(N – N)] cm^-1^.

### S3. Refinement

The H atom bonded to N3 was located in a difference Fourier map and refined freely; N3–H3N3 = 1.02 (3) Å. Other H atoms were positioned geometrically and allowed to ride on their parent atoms, with C—H = 0.93 Å, and with *U*_iso_(H) = 1.2*U*_eq_(C). The ISOR restraint and EADP constraint commands in the *SHELXL2014* software were used for the disordered atoms.

### Crystal data

**Table:**

C_12_H_9_N_3_O_2_	*F*(000) = 472
*M**_r_* = 227.22	*D*_x_ = 1.424 Mg m^−^^3^
Monoclinic, *P*2_1_/*n*	Mo *K*α radiation, λ = 0.71073 Å
*a* = 13.195 (3) Å	Cell parameters from 594 reflections
*b* = 5.6162 (8) Å	θ = 3.1–29.0°
*c* = 14.958 (3) Å	µ = 0.10 mm^−^^1^
β = 107.00 (2)°	*T* = 293 K
*V* = 1060.0 (3) Å^3^	Needle, colourless
*Z* = 4	0.4 × 0.3 × 0.15 mm

### Data collection

**Table:**

Agilent Xcalibur Eos diffractometer	2405 independent reflections
Radiation source: Enhance (Mo) X-ray Source	1057 reflections with *I* > 2σ(*I*)
Graphite monochromator	*R*_int_ = 0.039
Detector resolution: 16.0938 pixels mm^-1^	θ_max_ = 29.1°, θ_min_ = 3.2°
ω scans	*h* = −18→13
Absorption correction: multi-scan (*CrysAlis PRO*; Agilent, 2011)	*k* = −7→7
*T*_min_ = 0.941, *T*_max_ = 1.000	*l* = −19→17
4305 measured reflections	

### Refinement

**Table:**

Refinement on *F*^2^	Primary atom site location: structure-invariant direct methods
Least-squares matrix: full	Secondary atom site location: difference Fourier map
*R*[*F*^2^ > 2σ(*F*^2^)] = 0.064	Hydrogen site location: mixed
*wR*(*F*^2^) = 0.142	H atoms treated by a mixture of independent and constrained refinement
*S* = 1.01	*w* = 1/[σ^2^(*F*_o_^2^) + (0.0308*P*)^2^] where *P* = (*F*_o_^2^ + 2*F*_c_^2^)/3
2405 reflections	(Δ/σ)_max_ < 0.001
174 parameters	Δρ_max_ = 0.19 e Å^−^^3^
40 restraints	Δρ_min_ = −0.21 e Å^−^^3^

### Special details

**Table:**

Geometry. All e.s.d.'s (except the e.s.d. in the dihedral angle between two l.s. planes) are estimated using the full covariance matrix. The cell e.s.d.'s are taken into account individually in the estimation of e.s.d.'s in distances, angles and torsion angles; correlations between e.s.d.'s in cell parameters are only used when they are defined by crystal symmetry. An approximate (isotropic) treatment of cell e.s.d.'s is used for estimating e.s.d.'s involving l.s. planes.
Refinement. Refinement on *F*^2^ for ALL reflections except those flagged by the user for potential systematic errors. Weighted *R*-factors *wR* and all goodnesses of fit *S* are based on *F*^2^, conventional *R*-factors *R* are based on *F*, with *F* set to zero for negative *F*^2^. The observed criterion of *F*^2^ > σ(*F*^2^) is used only for calculating -*R*-factor-obs *etc*. and is not relevant to the choice of reflections for refinement. *R*-factors based on *F*^2^ are statistically about twice as large as those based on *F*, and *R*-factors based on ALL data will be even larger.

### Fractional atomic coordinates and isotropic or equivalent isotropic displacement parameters (Å^2^)

**Table:**

	*x*	*y*	*z*	*U*_iso_*/*U*_eq_	Occ. (<1)
O2	0.65450 (17)	0.5460 (3)	0.46696 (13)	0.0425 (6)	
N1	0.6233 (2)	0.5577 (4)	0.60530 (17)	0.0463 (7)	
N2	0.5682 (2)	0.7470 (4)	0.54975 (17)	0.0445 (7)	
N3	0.5544 (2)	0.8799 (4)	0.39788 (17)	0.0449 (7)	
H3N3	0.512 (2)	1.014 (5)	0.4150 (19)	0.067 (11)*	
O1	0.7733 (7)	0.1580 (16)	0.5086 (4)	0.0560 (14)	0.76 (2)
C1	0.8374 (6)	−0.0405 (14)	0.5478 (9)	0.057 (2)	0.76 (2)
H1A	0.8716	−0.1392	0.5158	0.068*	0.76 (2)
C2	0.8412 (7)	−0.0643 (15)	0.6370 (8)	0.062 (2)	0.76 (2)
H2A	0.8780	−0.1815	0.6776	0.074*	0.76 (2)
C3	0.7797 (8)	0.1194 (18)	0.6598 (6)	0.0470 (17)	0.76 (2)
H3A	0.7692	0.1477	0.7177	0.056*	0.76 (2)
C4	0.740 (2)	0.244 (4)	0.5812 (9)	0.0395 (13)	0.76 (2)
O1A	0.790 (3)	0.093 (5)	0.5177 (17)	0.0560 (14)	0.24 (2)
C1A	0.844 (3)	−0.073 (5)	0.587 (2)	0.057 (2)	0.24 (2)
H1AA	0.8868	−0.1956	0.5766	0.068*	0.24 (2)
C2A	0.826 (3)	−0.029 (5)	0.6672 (18)	0.062 (2)	0.24 (2)
H2AA	0.8545	−0.1113	0.7226	0.074*	0.24 (2)
C3A	0.755 (3)	0.163 (6)	0.655 (2)	0.0470 (17)	0.24 (2)
H3AA	0.7244	0.2242	0.6989	0.056*	0.24 (2)
C4A	0.741 (8)	0.238 (13)	0.568 (3)	0.0395 (13)	0.24 (2)
C5	0.6717 (3)	0.4467 (5)	0.5543 (2)	0.0411 (8)	
C6	0.5901 (3)	0.7321 (5)	0.4705 (2)	0.0409 (8)	
C7	0.5742 (3)	0.8830 (5)	0.3105 (2)	0.0408 (8)	
C8	0.5304 (3)	1.0704 (5)	0.2521 (2)	0.0515 (9)	
H8A	0.4916	1.1860	0.2722	0.062*	
C9	0.5436 (3)	1.0878 (6)	0.1647 (2)	0.0627 (11)	
H9A	0.5129	1.2139	0.1259	0.075*	
C10	0.6013 (3)	0.9215 (6)	0.1342 (2)	0.0607 (11)	
H10A	0.6097	0.9332	0.0748	0.073*	
C11	0.6470 (3)	0.7360 (6)	0.1925 (2)	0.0593 (10)	
H11A	0.6876	0.6238	0.1727	0.071*	
C12	0.6330 (3)	0.7158 (5)	0.2798 (2)	0.0518 (9)	
H12A	0.6633	0.5889	0.3183	0.062*	

### Atomic displacement parameters (Å^2^)

**Table:**

	*U*^11^	*U*^22^	*U*^33^	*U*^12^	*U*^13^	*U*^23^
O2	0.0432 (14)	0.0445 (12)	0.0412 (12)	0.0048 (11)	0.0147 (11)	0.0035 (9)
N1	0.0496 (18)	0.0517 (15)	0.0372 (15)	−0.0012 (15)	0.0123 (14)	−0.0020 (12)
N2	0.0463 (18)	0.0501 (16)	0.0384 (15)	0.0039 (14)	0.0146 (14)	0.0015 (12)
N3	0.0511 (19)	0.0456 (16)	0.0418 (16)	0.0102 (14)	0.0192 (15)	0.0040 (12)
O1	0.061 (3)	0.044 (4)	0.065 (2)	0.017 (3)	0.022 (2)	0.006 (2)
C1	0.049 (3)	0.036 (3)	0.081 (6)	0.014 (2)	0.014 (4)	0.012 (4)
C2	0.060 (4)	0.064 (3)	0.055 (5)	0.001 (3)	0.007 (4)	0.014 (4)
C3	0.043 (5)	0.048 (4)	0.042 (2)	0.017 (3)	−0.001 (3)	0.017 (2)
C4	0.042 (2)	0.043 (2)	0.035 (4)	−0.0025 (17)	0.013 (5)	−0.002 (4)
O1A	0.061 (3)	0.044 (4)	0.065 (2)	0.017 (3)	0.022 (2)	0.006 (2)
C1A	0.049 (3)	0.036 (3)	0.081 (6)	0.014 (2)	0.014 (4)	0.012 (4)
C2A	0.060 (4)	0.064 (3)	0.055 (5)	0.001 (3)	0.007 (4)	0.014 (4)
C3A	0.043 (5)	0.048 (4)	0.042 (2)	0.017 (3)	−0.001 (3)	0.017 (2)
C4A	0.042 (2)	0.043 (2)	0.035 (4)	−0.0025 (17)	0.013 (5)	−0.002 (4)
C5	0.042 (2)	0.0453 (18)	0.0321 (17)	−0.0056 (17)	0.0056 (16)	0.0040 (14)
C6	0.039 (2)	0.0412 (18)	0.0430 (19)	−0.0004 (16)	0.0122 (16)	−0.0018 (15)
C7	0.041 (2)	0.0437 (18)	0.0377 (18)	−0.0019 (17)	0.0112 (16)	0.0004 (14)
C8	0.062 (2)	0.0446 (19)	0.052 (2)	0.0121 (18)	0.023 (2)	0.0051 (16)
C9	0.079 (3)	0.062 (2)	0.052 (2)	0.020 (2)	0.026 (2)	0.0165 (17)
C10	0.073 (3)	0.070 (2)	0.047 (2)	0.010 (2)	0.030 (2)	0.0091 (18)
C11	0.070 (3)	0.061 (2)	0.056 (2)	0.013 (2)	0.033 (2)	−0.0006 (18)
C12	0.058 (2)	0.0509 (19)	0.051 (2)	0.0180 (19)	0.0230 (19)	0.0114 (16)

### Geometric parameters (Å, º)

**Table:**

O2—C6	1.358 (3)	C1A—C2A	1.321 (16)
O2—C5	1.376 (3)	C1A—H1AA	0.9300
N1—C5	1.290 (4)	C2A—C3A	1.397 (16)
N1—N2	1.413 (3)	C2A—H2AA	0.9300
N2—C6	1.302 (4)	C3A—C4A	1.335 (16)
N3—C6	1.339 (3)	C3A—H3AA	0.9300
N3—C7	1.406 (4)	C4A—C5	1.46 (2)
N3—H3N3	1.02 (3)	C7—C12	1.379 (4)
O1—C4	1.375 (6)	C7—C8	1.382 (4)
O1—C1	1.418 (6)	C8—C9	1.371 (4)
C1—C2	1.328 (7)	C8—H8A	0.9300
C1—H1A	0.9300	C9—C10	1.365 (4)
C2—C3	1.414 (7)	C9—H9A	0.9300
C2—H2A	0.9300	C10—C11	1.379 (4)
C3—C4	1.337 (6)	C10—H10A	0.9300
C3—H3A	0.9300	C11—C12	1.376 (4)
C4—C5	1.433 (8)	C11—H11A	0.9300
O1A—C4A	1.387 (16)	C12—H12A	0.9300
O1A—C1A	1.418 (16)		
			
C6—O2—C5	101.9 (2)	C3A—C4A—O1A	112.9 (16)
C5—N1—N2	105.9 (2)	C3A—C4A—C5	107 (2)
C6—N2—N1	105.9 (3)	O1A—C4A—C5	140 (3)
C6—N3—C7	130.2 (3)	N1—C5—O2	113.1 (3)
C6—N3—H3N3	110.1 (16)	N1—C5—C4	126.5 (5)
C7—N3—H3N3	119.5 (16)	O2—C5—C4	120.3 (5)
C4—O1—C1	103.9 (5)	N1—C5—C4A	134.6 (14)
C2—C1—O1	109.7 (5)	O2—C5—C4A	112.2 (13)
C2—C1—H1A	125.1	N2—C6—N3	125.4 (3)
O1—C1—H1A	125.1	N2—C6—O2	113.2 (3)
C1—C2—C3	108.3 (5)	N3—C6—O2	121.4 (3)
C1—C2—H2A	125.9	C12—C7—C8	118.7 (3)
C3—C2—H2A	125.9	C12—C7—N3	125.2 (3)
C4—C3—C2	106.1 (5)	C8—C7—N3	116.1 (3)
C4—C3—H3A	127.0	C9—C8—C7	120.7 (3)
C2—C3—H3A	127.0	C9—C8—H8A	119.6
C3—C4—O1	112.1 (6)	C7—C8—H8A	119.6
C3—C4—C5	135.8 (8)	C10—C9—C8	120.6 (3)
O1—C4—C5	112.1 (8)	C10—C9—H9A	119.7
C4A—O1A—C1A	102.0 (14)	C8—C9—H9A	119.7
C2A—C1A—O1A	110.6 (15)	C9—C10—C11	119.2 (3)
C2A—C1A—H1AA	124.7	C9—C10—H10A	120.4
O1A—C1A—H1AA	124.7	C11—C10—H10A	120.4
C1A—C2A—C3A	108.7 (16)	C12—C11—C10	120.5 (3)
C1A—C2A—H2AA	125.6	C12—C11—H11A	119.8
C3A—C2A—H2AA	125.6	C10—C11—H11A	119.8
C4A—C3A—C2A	105.5 (16)	C11—C12—C7	120.3 (3)
C4A—C3A—H3AA	127.3	C11—C12—H12A	119.9
C2A—C3A—H3AA	127.3	C7—C12—H12A	119.9
			
C5—N1—N2—C6	−0.7 (3)	C3—C4—C5—O2	173 (3)
C4—O1—C1—C2	−0.7 (18)	O1—C4—C5—O2	−6 (3)
O1—C1—C2—C3	−0.2 (11)	C3A—C4A—C5—N1	−2 (12)
C1—C2—C3—C4	1.1 (19)	O1A—C4A—C5—N1	173 (9)
C2—C3—C4—O1	−2 (3)	C3A—C4A—C5—O2	178 (5)
C2—C3—C4—C5	179 (3)	O1A—C4A—C5—O2	−7 (15)
C1—O1—C4—C3	1 (3)	N1—N2—C6—N3	−179.4 (3)
C1—O1—C4—C5	−179.2 (18)	N1—N2—C6—O2	0.9 (3)
C4A—O1A—C1A—C2A	1 (7)	C7—N3—C6—N2	178.8 (3)
O1A—C1A—C2A—C3A	2 (5)	C7—N3—C6—O2	−1.5 (5)
C1A—C2A—C3A—C4A	−5 (7)	C5—O2—C6—N2	−0.7 (3)
C2A—C3A—C4A—O1A	6 (10)	C5—O2—C6—N3	179.6 (3)
C2A—C3A—C4A—C5	−177 (5)	C6—N3—C7—C12	4.1 (5)
C1A—O1A—C4A—C3A	−5 (9)	C6—N3—C7—C8	−175.8 (3)
C1A—O1A—C4A—C5	−180 (12)	C12—C7—C8—C9	1.1 (5)
N2—N1—C5—O2	0.2 (3)	N3—C7—C8—C9	−178.9 (3)
N2—N1—C5—C4	178.7 (18)	C7—C8—C9—C10	−0.8 (6)
N2—N1—C5—C4A	−180 (7)	C8—C9—C10—C11	−0.4 (6)
C6—O2—C5—N1	0.3 (3)	C9—C10—C11—C12	1.3 (6)
C6—O2—C5—C4	−178.3 (17)	C10—C11—C12—C7	−0.9 (6)
C6—O2—C5—C4A	−180 (5)	C8—C7—C12—C11	−0.3 (5)
C3—C4—C5—N1	−5 (5)	N3—C7—C12—C11	179.8 (3)
O1—C4—C5—N1	175.9 (13)		

### Hydrogen-bond geometry (Å, º)

Cg1 and Cg4 are the centroids of the O1*A*/C1/C2/C3/C4 and C7–C12 five- and six-membered rings, respectively.

**Table:**

*D*—H···*A*	*D*—H	H···*A*	*D*···*A*	*D*—H···*A*
N3—H3*N*3···N2^i^	1.02 (3)	1.87 (3)	2.892 (3)	178 (2)
C12—H12*A*···O2	0.93	2.27	2.892 (4)	123
C9—H9*A*···*Cg*1^ii^	0.93	3.00	3.653 (4)	129
C2—H2*A*···*Cg*4^iii^	0.93	2.93	3.664 (5)	137

Symmetry codes: (i) −*x*+1, −*y*+2, −*z*+1; (ii) *x*−1/2, −*y*−1/2, *z*−1/2; (iii) *x*−3/2, −*y*+1/2, *z*−3/2.
