# Supplementary figures and images for: Crystal structure of 5-(furan-2-yl)-N-phenyl-1,3,4-oxa­diazol-2-amine
Source: Acta Crystallogr E Crystallogr Commun. 2015 Oct 24;71(Pt 11):o880–1. doi: 10.1107/S2056989015019453 (PMC4645015; doi:10.1107/S2056989015019453)

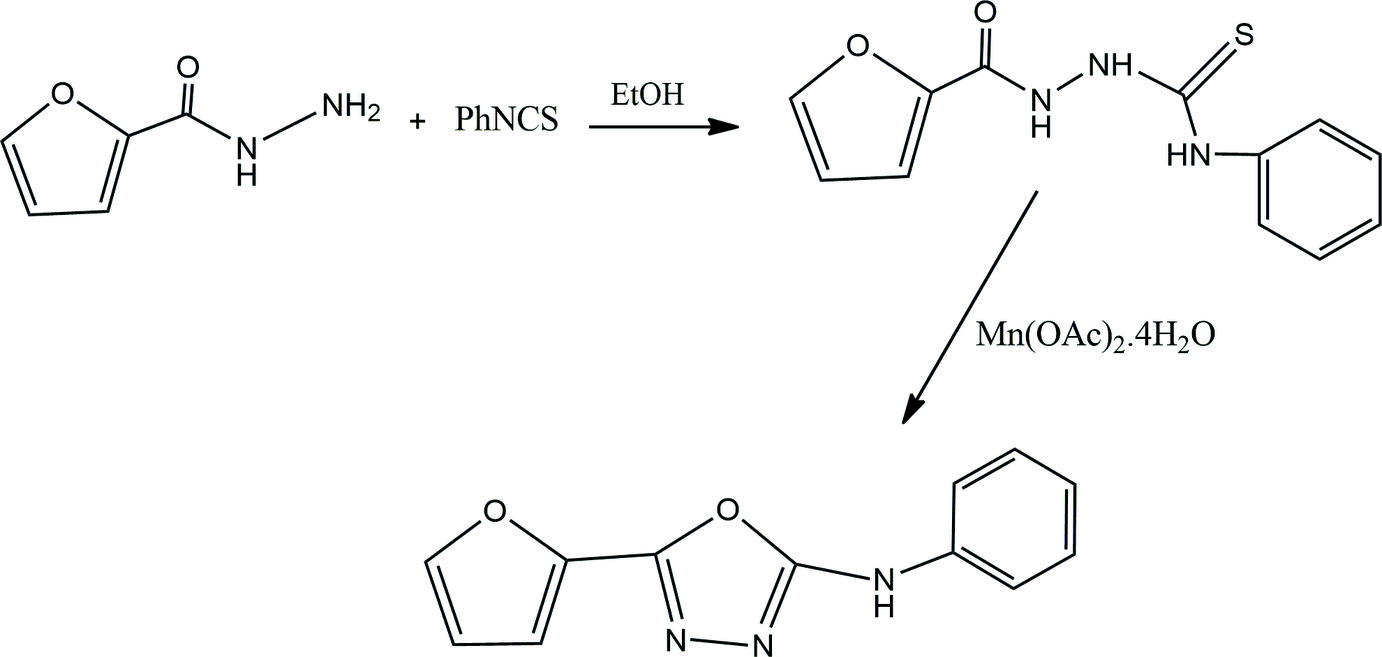

Supplement: Supplementary file 4 [file e-71-0o880-fig1.tif]

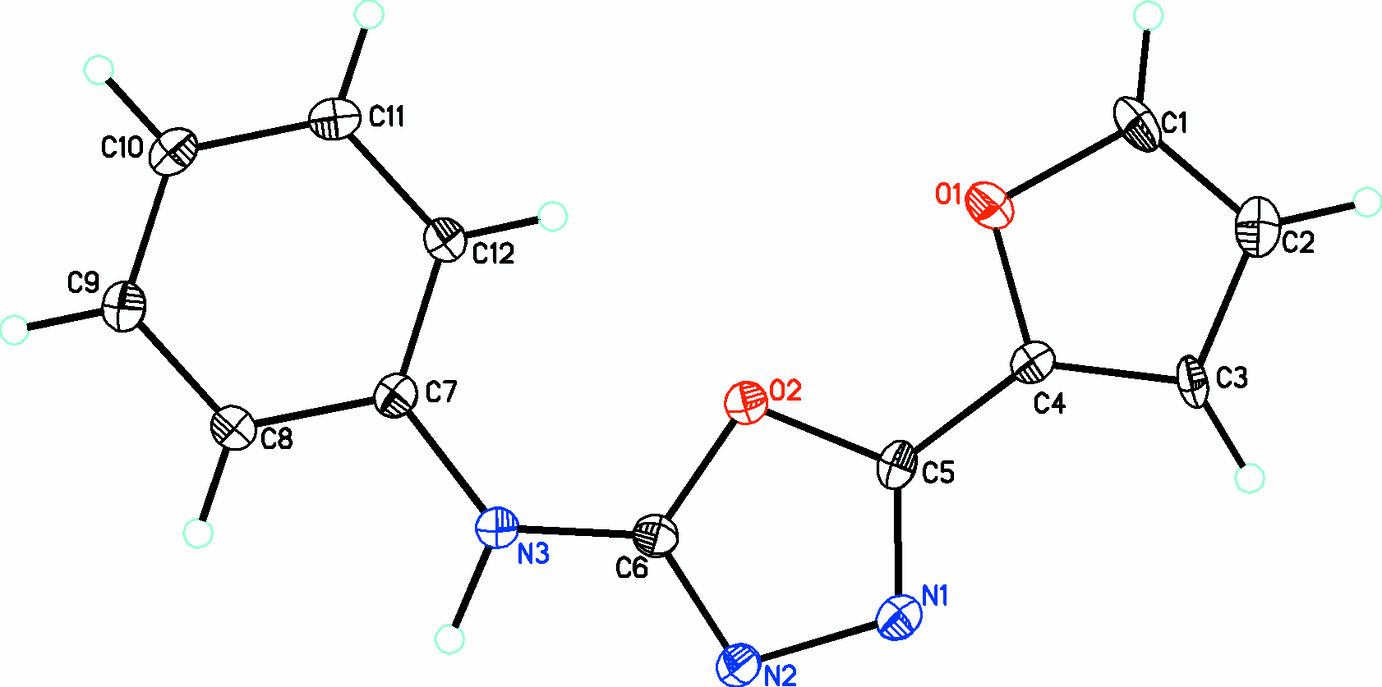

Supplement: Supplementary file 5 [file e-71-0o880-fig2.tif]

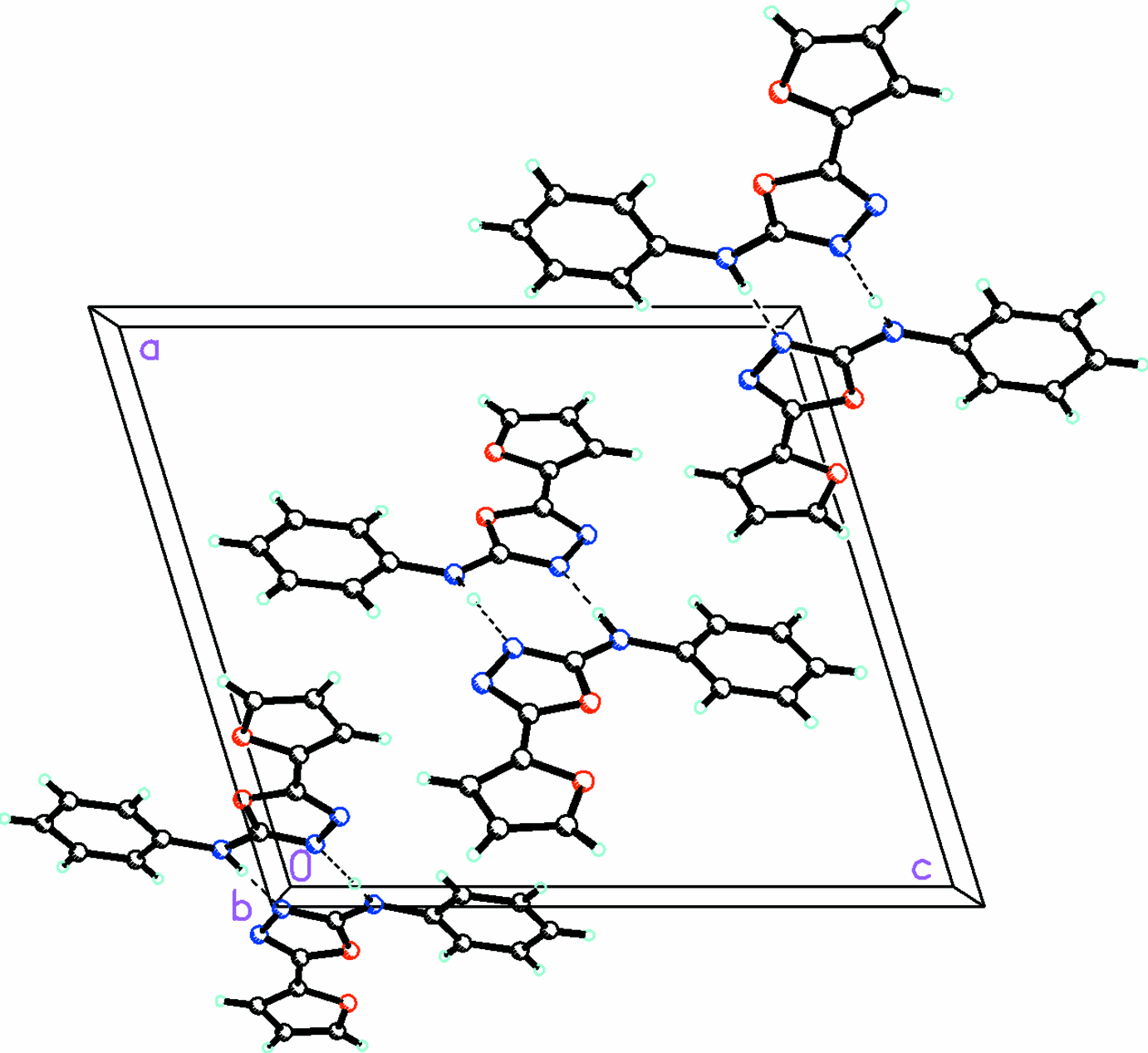

Supplement: Supplementary file 6 [file e-71-0o880-fig3.tif]
